# Morfofunctional and Molecular Changes in Placenta and Peripheral Blood in Preeclampsia and Gestational Diabetes Mellitus

**DOI:** 10.1134/S0012496623700722

**Published:** 2023-12-08

**Authors:** K. A. Artemieva, Yu. V. Stepanova, I. I. Stepanova, M. V. Shamarakova, N. B. Tikhonova, N. V. Nizyaeva, S. G. Tsakhilova, L. M. Mikhaleva

**Affiliations:** 1 Avtsyn Institute of Human Morphology, Petrovsky National Research Centre of Surgery, Moscow, Russia; 2 Evdokimov Moscow State University of Medicine and Dentistry, Moscow, Russia; 3 Yudin City Clinical Hospital, Moscow Healthcare Department, Moscow, Russia

**Keywords:** pregnancy, preeclampsia, gestational diabetes mellitus, placental proteins, inflammation

## Abstract

Gestational diabetes mellitus (GDM) and preeclampsia (PE) are common pregnancy complications with similar risk factors. Although GDM is associated with PE, the exact mechanism underlying the association is unclear. The objective of this work was to study the morphofunctional and molecular changes in the placenta and peripheral blood in PE and GDM. Local and systemic changes in the production of several placental proteins were assessed along with markers of inflammation and metabolic disorders. Expression of placental lactogen, trophoblastic β1-glycoprotein, placental alpha-1-microglobulin, and proteinase 3 in villi was found to change in complicated pregnancy groups. Similarity of underlying pathogenic mechanisms was demonstrated for PE and GDM.

## INTRODUCTION

Gestational diabetes mellitus (GDM) and preeclampsia (PE) are common pregnancy complications with similar risk factors (obesity, age over 35, multiparity, etc.) [1]. GDM is defined as glucose intolerance first diagnosed during pregnancy. GDM is associated with obstetric and neonatal complications and is recognized as a risk factor for cardiometabolic disorders in the mother and offspring in the future. The PE incidence has also been shown to substantially increase in GDM [2].

PE is characterized by de novo hypertension (a systolic or diastolic blood pressure ≥140 or ≥90 mm Hg, respectively) diagnosed after 20 weeks of gestation with proteinuria and/or dysfunction of at least one organ (the kidney, liver, nervous system, etc.) and is a main cause of maternal and fetal morbidity and mortality [3]. GDM complicated by PE further increases perinatal adverse events and future maternal risk of hypertension, cardiovascular disorders, and diabetes mellitus [3–5].

Both in GDM and in PE, pathophysiological processes include oxidative stress, an enhanced inflammatory response, and endothelial dysfunction, leading to inadequate perfusion and hypoxia of the placenta [6].

Although GDM is associated with PE, the exact mechanism underlying the association is unclear. The PE pathophysiological process includes early insufficient trophoblast invasion, which leads to incomplete spiral artery remodeling and eventually causes placental ischemia and oxidative stress. Hyperglycemia can cause inflammation and autophagy of trophoblast cells and inhibit their migration and invasion.

Neutrophils are overactive in GDM and PE and release excessive amounts of neutrophil extracellular traps (NETs), including DNA fibers and serine proteases (cathepsin G, neutrophil elastase (NE), and proteinase 3 (PR3)), in inflammation sites. The proteases have recently been shown to activate proinflammatory cytokines, including interleukin (IL) 1β and TNF-α. Excessive NET production hinders circulation in the intervillous space, leading to placental ischemia [1, 7].

Local placental regulatory factors are of special importance for crosstalk between the fetal and maternal compartments at the fetoplacental boundary and the control of the growth, differentiation, and functions of the placenta. Placental hormones can additionally affect distant maternal targets, acting to facilitate adaptation of maternal circulation to pregnancy, hemotrophic nutrition of the fetus, and the development and function of the mammary gland [8]. Placental lactogen (PL) is a peptide hormone and is secreted throughout pregnancy. PL plays an important role in regulating insulin secretion in pancreatic β cells. Dysregulation of PL secretion can promote placental dysfunction and diabetic retinopathy, cause fetal growth restriction or macrosomia, and affect the metabolic status in adulthood [8].

Pregnancy-specific β1-glycoprotein (PSG) provides one of the most informative markers of the formation and function of the fetoplacental system. PSG has been shown to contribute to the development of immune tolerance during pregnancy by increasing the Treg level, stimulating IL-10 production in the Treg subpopulation, and simultaneously suppressing IL-17A production in the Th17 subpopulation [9].

Placental α-1-macroglobulin (PAMG, insulin-like growth factor-binding protein 1 (IGFBP-1)) affects cell growth and metabolism. PAMG is involved in implantation and fetal growth and thus plays an important local role during pregnancy. Higher PAMG concentrations have been observed in PE, PAMG production occurring most likely in both decidua and liver [10].

Adipokines are adipose tissue hormones and are responsible for regulating certain physiological functions. Adiponectin is an important adipokine. Adiponectin secretion is stimulated by insulin. Adiponectin is involved in regulating the glucose level and fatty acid decomposition. It is thought that adiponectin exerts a protective effect against hyperglycemia, insulin resistance, and atherosclerosis.

Adiponectin directly affects the function of the placenta by modulating the transport of nutrients to the fetus via complex interactions between insulin signal transmission, insulin signal responses, and the cytokine profile [11]. Lack of a perfect marker to predict PE in women with GDM renders it necessary to comprehensively study the pathophysiology of GDM and PE. There is no test now to reliably detect placental dysfunction in late pregnancy.

The objective of this work was to study the morphofunctional and molecular changes in the placenta and peripheral blood in PE and GDM.

## MATERIALS AND METHODS

The study included pregnant women with GDM (*n* = 35), PE (*n* = 35), or PE combined with GDM (*n* = 30) and women with uncomplicated physiolo-gical pregnancy (PP) (*n* = 30) matched for gestatio-nal age.

All patients provided their voluntary informed consent for participation in the study.

Inclusion criteria for the PE group:

1. Singleton pregnancy.

2. Pregnant women aged 18–40 years.

3. Positive PE criteria (arterial hypertension (blood pressure ≥140/90 mm Hg), proteinuria (>0.3 g/L in  24-h urine), edemas, and signs of multiple organ failure).

4. Delivery by cesarean section.

Inclusion criteria for the PP group:

1. PP from a natural cycle.

2. Surgical delivery according to obstetric indications.

3. Protein urine level <100 µg/mL.

4. No sign of arterial hypertension.

5. Fasting venous plasma glucose <5.1 mmol/L.

Inclusion criteria for the GCM group comprised fasting venous plasma glucose ≥5.1, but <7.0 mmol/L at any gestational age; in particular, 1-h plasma glucose ≥10.0 mmol/L and 2-h plasma glucose ≥8.5, but <11.1 mmol/L in the oral glucose tolerance test [12].

The PE + GMD group included the pregnant patients with combined signs of the PE and GDM groups.

**Histological examination.** Placental tissue samples (1.5 × 1.5 × 1 cm) were taken from the marginal, paracentral, and central regions of the placental disc. The samples were fixed with 10% formalin (pH 7.4) (Biovitrum, Russia) for 24 h, embedded in paraffin, and used to obtain 4-µm sections. At least 10 samples were examined.

**Immunohistochemistry** (IHC) was carried out using placental paraffin sections. Mouse monoclonal antibodies (ABs) to PAMG, PSG, PL, and PR3 were used as primary ABs. The ABs were obtained in the Laboratory of Reproductive Pathology (Avtsyn Institute of Human Morphology) as described by Starosvetskaya et al. [13]. A PrimeVision two-component detection system (PrimeBioMed, Russia) was used. After incubation, the sections were stained with Mayer’s hematoxylin solution. As a negative control, the IHC protocol was carried out without using primary ABs. The ICH staining intensity was measured in optical density units (ODUs), using a Leica DM 2500 light microscope with a digital camera, a graphic tablet, and ImageScopeM software (Leica Microsystems GmbH, Germany). Twenty fields of vision were examined in each preparation at magnification ×400. The optical densities of the background (empty slide) and negative control were subtracted.

**Enzyme-linked immunosorbent assay** (ELISA) was performed for PL, human PSG, and PAMG with commercial kits as recommended by the manufacturer (Diatekh-EM, Russia). The adiponectin and PR3 concentrations were measured by quantitative sandwich ELISA, using 96-well polystyrene plates (Maxibinding, SPL Life Sciences, Seoul, South Korea) and mouse monoclonal ABs (clones PN17/PN17 (adiponectin) and PR49/PR45 (PR3)), which were obtained from the Laboratory of Reproductive Pathology (Avtsyn Institute of Human Morphology).

**Statistical analyses** of the results were carried out using Sigma Stat 3.5 (Systat Software). Parameter distributions were tested for normality by the Kolmogorov–Smirnov test. Differences between groups were evaluated by the paired Mann–Whitney test. The results were expressed as the median (Me) and first and third quartiles (Q1 and Q3) Differences were considered significant at *p* < 0.05; *p* < 0.1 suggested a trend.

## RESULTS AND DISCUSSION

As ELISA results showed (Table 1), the PAMG and PL concentrations in the blood in all test groups were higher than in the control PP group. PSG levels did not significantly differ between groups. The adiponectin level was the lowest in the PP group and showed no between-group difference. The PR3 content was the highest in the PP group, in contrast to local PR3 detection in villi.

**Table 1.** Levels of the placental proteins, PR3, and adiponectin in the blood plasma

	PAMG, ng/mL	PSG, µg/mL	PL, mg/L	PR3, ng/mL	Adiponectin, µg/mL
PP *1*	29.35 (24.65;34.4)	246.3 (136.93;630.68)	0.9 (0.15;1.3)	21.45 (15.08;51)	10.99 (6.52;16.03)
GDM *2*	40.4 (19.5;52.8)	252.4 (85.3; 415.4)	1.09 (0.93;2.4)	10.6 (5.57;21.8)	5.7 (5.23;7.39)
PE *3*	58.9* (53.7;127.7)	266.5 (226.45; 431.3)	1.6 (0.8;2)	12.9 (6.1;36.8)	6.11 (4.75;7.42)
PE + GDM *4*	53.6 (36.6;84.08)	216.1 (171.05;317.25)	1.79 (0.8; 2.28)	7.85 (4.43;18.8)	6.24 (4.02;7.15)
Significance of differences	*p*1–2 = 0.469	*p*1–2 = 0.419	*p*1–2 = 0.056	*p*1–2 = 0.011	*p*1–2 = 0.019
	*p* 1–3 < 0.001	*p*1–3 = 0.982	*p*1–3 = 0.046	*p*1–3 = 0.02	*p*1–3 = 0.027
	*p* 1–4 = 0.002	*p*1–4 = 0.693	*p*1–4 = 0.026	*p*1–4 = 0.015	*p*1–4 = 0.007
	*p*2–4 = 0.032	*p*2–4 = 0.842	*p*2–4 = 0.602	*p*2–4 = 0.602	*p*2–4 = 0.552
	*p*3–4 = 0.177	*p*3–4 = 0.190	*p*3–4 = 0.819	*p*3–4 = 0.360	*p*3–4 = 0.643

**Fig. 1.**
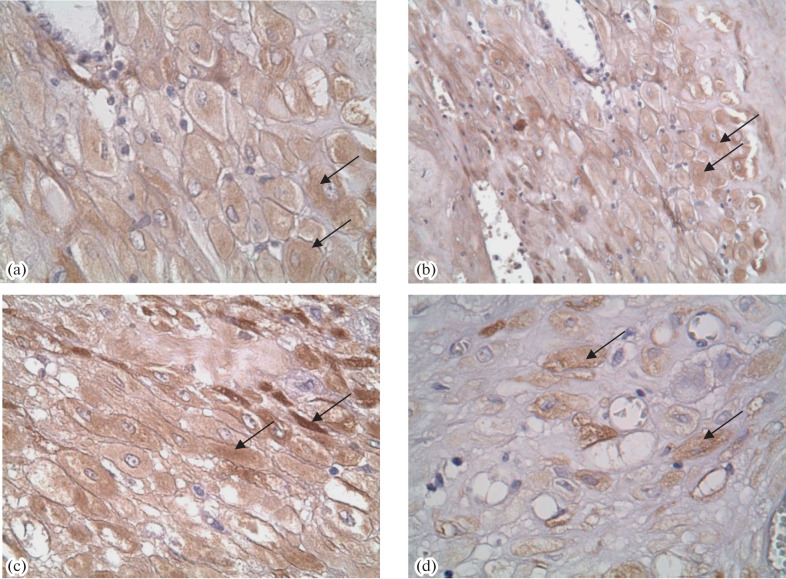
IHC staining of the placenta with AB to PAMG in the (a) PP, (b) GDM, (c) PE, and (d) PE + GDM groups. A positive staining of decidual cells is indicated with arrows. Magnificantion ×400.

**Fig. 2.**
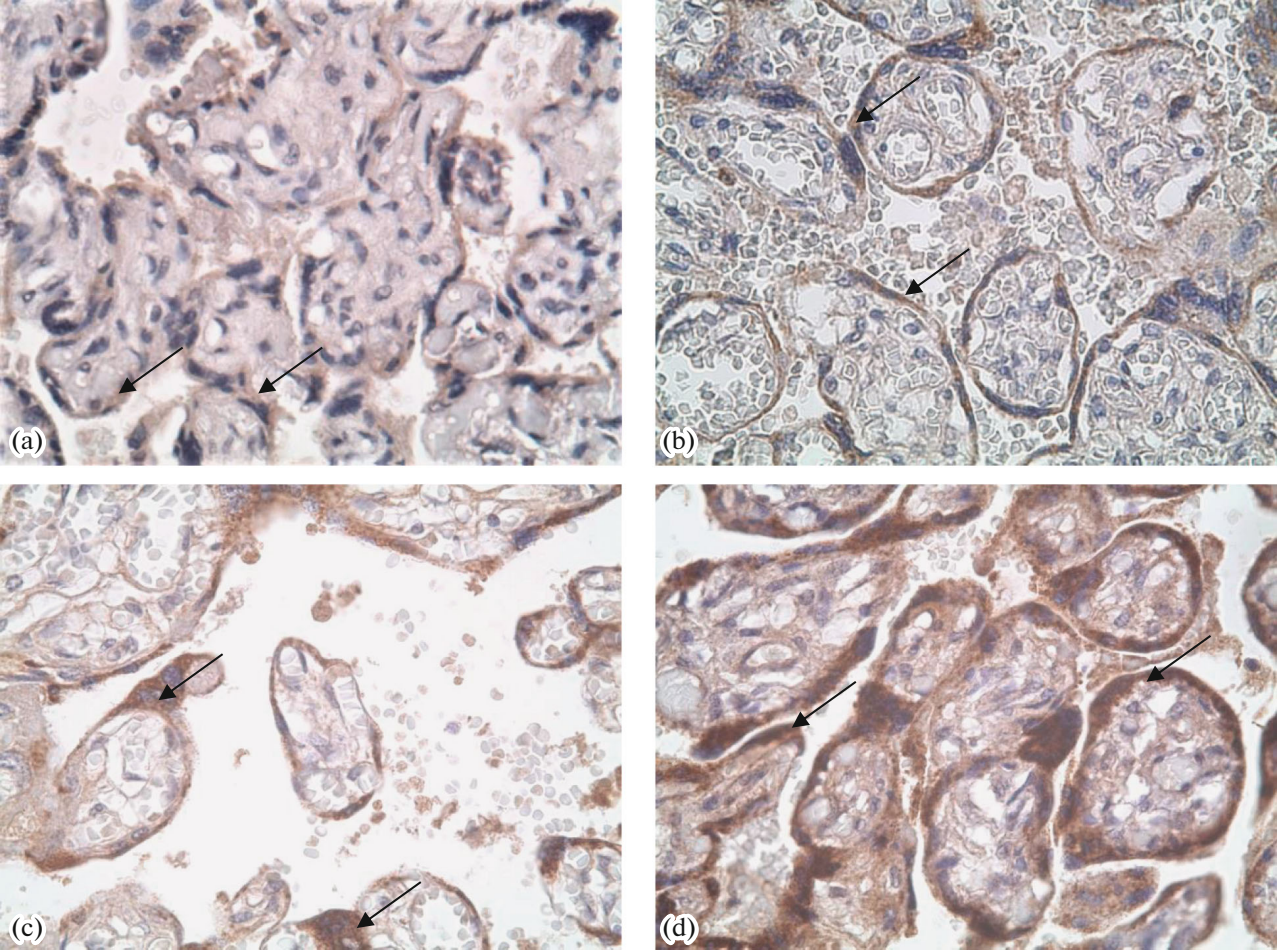
IHC staining of the placenta with AB to PSG in the (a) PP, (b) GDM, (c) PE, and (d) PE + GDM groups. A positive staining of the syncytiotrophoblast of placental villi is indicated with arrows. Magnificantion ×400.

**Fig. 3.**
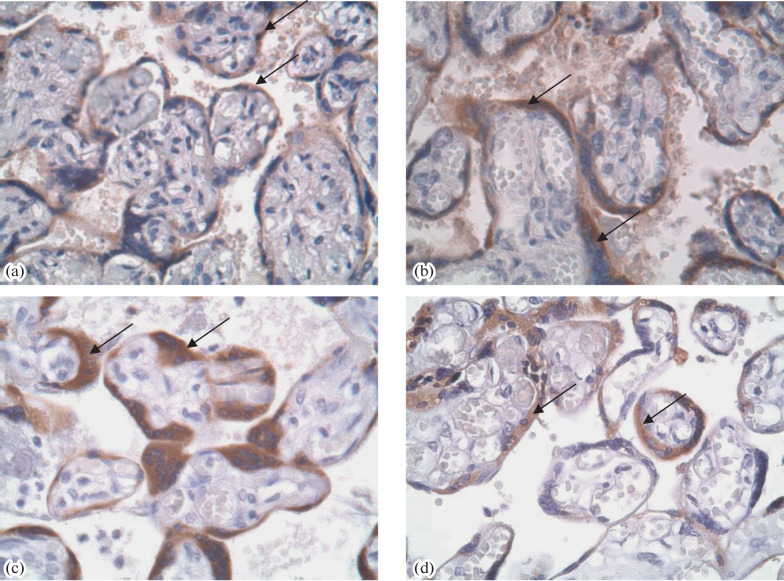
IHC staining of the placenta with AB to PL in the (a) PP, (b) GDM, (c) PE, and (d) PE + GDM groups. A positive staining of the syncytiotrophoblast of placental villi is indicated with arrows. Magnificantion ×400.

**Fig. 4.**
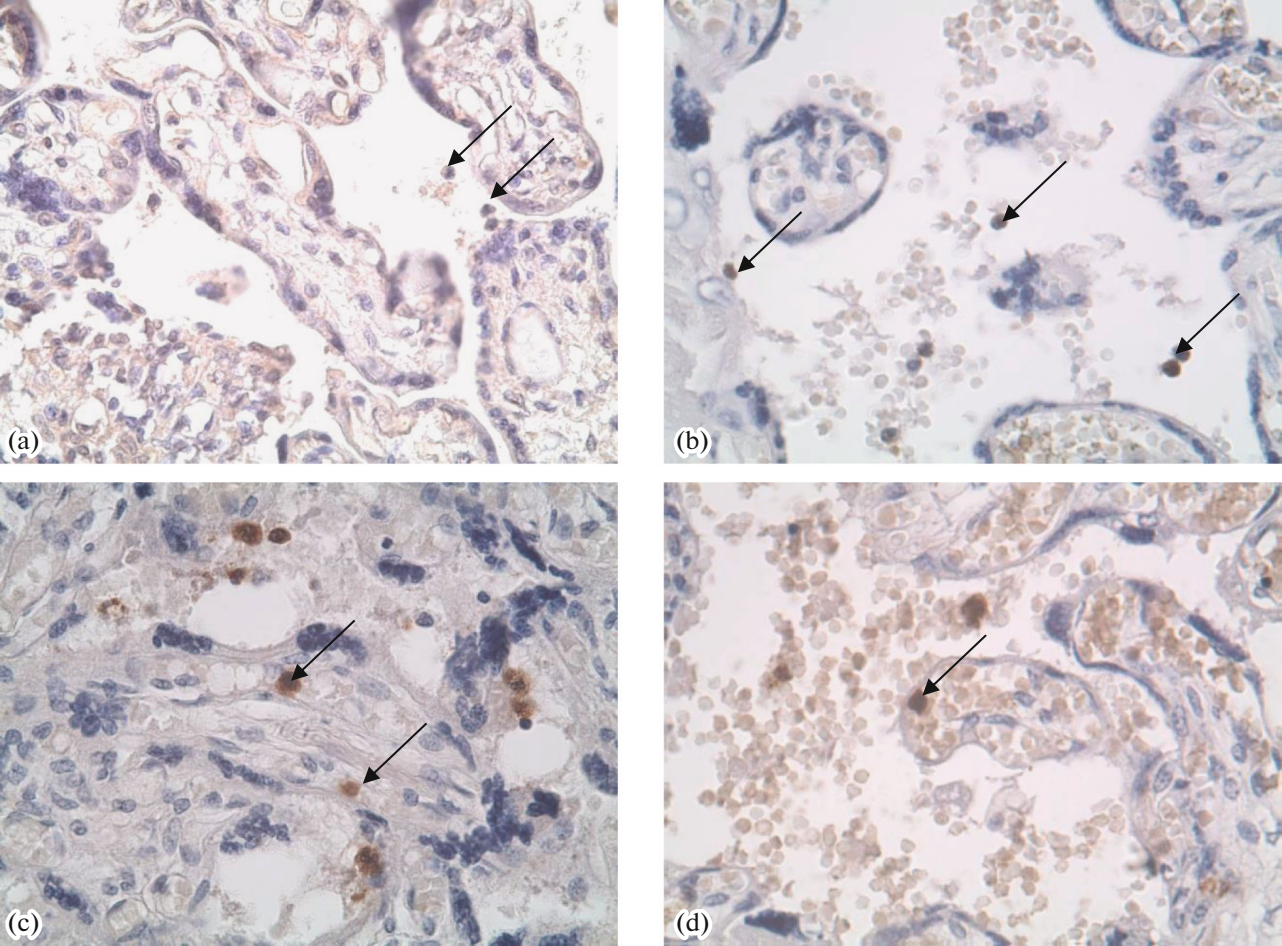
IHC staining of the placenta with AB to PR3 in the (a) PP, (b) GDM, (c) PE, and (d) PE + GDM groups. A positive staining of neutrophils in placental villi is indicated with arrows. Magnificantion ×400.

In IHC, a more intense staining of villi with AB to PL was observed in the groups of pregnancies complicated with GDM, PE, or their combination as compared with the PP group (Table 2, Figs. 1–4). The staining intensities with AB to PAMG in the GDM and PE + GDM groups were lower than in the PP group (Fig. 1). Staining with AB to PSG was the most intense in the PE and PE + GDM groups (Fig. 2). Staining of villi with AB to PL was irregular, and trophoblast shedding into the intevillous space was observed (Fig. 3). AT to PR3 intensely stained the placental villi in all test groups compared with the PP group (Fig. 4).

**Table 2.** IHC staining intensity (ODU) of the placenta

	PAMG	PSG	PL	PR3
PP *1*	60.46 (53.95; 63.8)	56.82 (31.49;85.00)	35.05 (31.89;40.32)	31.88 (28.66;38.02)
GDM *2*	46.84 (38.17;63.07)	47.99 (42.96;70.79)	82.46 (60.50;93.76)	60.94 (50.75; 73.80)
PE *3*	75.49 (66.28;85.61)	71.76 (62.39;78.89)	70.10 (64.63;76.22)	61.67 (58.0;70.43)
PE + GDM *4*	40.88 (32.52;58.06)	84.11 (80.91;93.04)	70.20 (65.53;77.31)	64.42 (49.95; 73.11)
Significance of differences	*p*1–2 = 0.014	*p*1–2 = 0.894	*p*1–2 < 0.001	*p*1–2 < 0.001
	*p*1–3 < 0.001	*p*1–3 = 0.374	*p*1–3 < 0.001	*p*1–3 < 0.001
	*p*1–4 = 0.017	*p*1–4 = 0.017	*p*1–4 < 0.001	*p*1–4 = 0.009
	*p*2–4 = 0.519	*p*2–4 < 0.001	*p*2–4 = 0.081	*p*2–4 = 0.894
	*p*3–4 = 0.009	*p*3–4 = 0.004	*p*3–4 = 0.963	*p*3–4 = 0.736

It should be noted that PE is difficult to diagnose in women with proteinuria documented before pregnancy [2]. This circumstance provided a substantial obstacle to testing in the PE and GDM groups. Discrepant data are available for PAMG expression in decidual cells in PE. It has been reported that PAMG in the placenta is elevated in PE and that PAMG overexpression inhibits proliferation, invasion, and migration in cultured HTR-8/SVneo cells [14]. At the same time, PAMG of the decidual plate stimulates extravillous trophoblast migration by binding with integrin α5β1 independently of the insulin-like growth factor (IGF) in the mother–fetus system [8]. Maternal hyperinsulinemia can suppress PAMG secretion from decidual cells, thus decreasing extravillous trophoblast migration and causing placental dysfunction. In our study, local PAMG expression in the placenta was decreased in the GDM and PE + GDM groups, while the PDGM concentration in the blood plasma was elevated in all groups with complicated pregnancy. The finding supports the hypothesis that PAMG plays a role in stimulating extravillous trophoblast migration; a decrease in PAMG expression is observed in the case of abnormal trophoblast invasion in PE [14].

PSG modulates extravillous trophoblast adhesion and migration by binding with integrin α5β1 [9]. The most intense staining with AB to PSG was detected in the PE and PE + GDM groups. It is clear that a local increase in PSG in the placenta is of compensatory nature in PE. In addition, PSG activates TGF-β1 and thereby facilitates the establishment of immune tolerance.

PL plays a predominant role in stimulating β-cell proliferation and insulin production in pregnancy and can be regulated by adiponectin [15]. It is important to note that vasoinhibin is generated via proteolytic cleavage of PL. Placental vasoinhibin may function to control the vascular growth in the placenta, but may also facilitate placental pathology in PE [16]. A higher PL concentration in the blood plasma and a higher PL-specific staining of placental villi were observed in women of all complicated pregnancy groups compared with the PP group. The finding supports the important role that PL plays in the pathogenesis of PE and GDM.

Adiponectin regulates trophoblast proliferation, differentiation, and invasion and modulates angiogenesis in the decidua by affecting secretion of IL-1β, IL-6, TNF-α, and PGE2 [17, 18]. The adiponectin level in the mother’s blood decreases from the first to the third trimester during uncomplicated pregnancy. Adiponectin prevents hypertension by suppressing the effects of angiotensin II in the renin–angiotensin–aldosterone system and helps to avert endothelial dysfunction, inflammation, and proteinuria [18]. In addition, a decrease in adiponectin expression correlates with insulin resistance and is associated with obesity [1]. In our study, the serum adiponectin levels in all complicated pregnancy groups were higher than in the PP group. Dysregulation of placental expression of adiponectin and its level in circulation may cause abnormal placentation and PE.

Neutrophils are known to promote vascular dysfunction in sepsis-induced inflammation. PR3 and NE, which are abundant in neutrophils, are released upon their degranulation [18]. A role of PR3 in endothelial dysfunction is poorly understood. Like NE, PR3 most likely causes dysfunction of the vascular barrier and increases the endothelial permeability [2, 7, 19]. A similar mechanism of PR3 possibly contributes to the pathogenesis of PE and GDM, manifesting itself in a local (placental) increase and a systemic decrease in PR3 in complicated pregnancy. A correlation between the IHC staining intensity and the protein level by ELISA was not assessed in this work. Such a correlation is not always absolute as a result of decompensated or compensatory local production of particular substances in the placenta. Another fact to consider is that certain proteins originate from both placental and extraplacental sources and can be synthesized in minor amounts in the liver, the lung, and cells arising via decidual transformation [20].

It is of interest to note that obesity exerts a lower effect on PE course in women with GDM as compared with women without GDM [1, 7]. The course of PE combined with GDM is more favorable than that of isolated PE by clinical signs and laboratory tests. Early distortions in carbohydrate metabolism can be assumed to suppress the pathological PE cascade by stimulating angiogenesis [21]. A substantial increase in the severity of patients’ conditions was not observed in the PE + GDM group in our study, thus supporting the above hypothesis.

## CONCLUSIONS

A similarity in the pathogenetic mechanisms of PE and GDM was observed in our study. We evaluated the local and systemic changes in the production of certain placental proteins that provide markers of inflammation and metabolic disorders. An increase in villus staining with ABs to PL and PR3 was observed in all test groups; a decrease in villus staining with AB to PAMG, in the GDM group; and an intense staining with AB to PSG, in the PE group. Elevated plasma concentrations of PSG, PL, and adiponectin were detected in all test groups. To improve pregnancy outcomes, it is necessary to further study and to identify the PE-associated factors in women with GDM. Optimization of GDM treatment and management can reduce the PE severity and incidence, thus also improving pregnancy outcomes.
